# Knowledge, attitudes, and practices about COVID-19 pandemic: a bibliometric analysis

**DOI:** 10.3389/fpubh.2023.1075729

**Published:** 2023-06-15

**Authors:** Laia Selva-Pareja, Carla Camí, Judith Roca, Anna Espart, Carme Campoy, Teresa Botigué

**Affiliations:** ^1^Department of Nursing and Physiotherapy, University of Lleida, Lleida, Spain; ^2^Càtedra de Desenvolupament i Territoris Saludables i Sostenibles (DOTSS), University of Lleida, Lleida, Spain; ^3^Health Education, Nursing, Sustainability and Innovation Research Group (GREISI), University of Lleida, Lleida, Spain; ^4^Health Care Research Group (GRECS), Biomedical Research Institute of Lleida, Lleida, Spain; ^5^Grup d’Estudis de Societats Africanes (GESA), University of Lleida, Lleida, Spain

**Keywords:** attitudes, bibliometric analysis, bibliometrix, COVID-19, knowledge, practices, VOSviewer

## Abstract

**Background:**

In order to avoid high rates of COVID-19 infection, one of the main tasks that must be performed is to improve the knowledge, attitudes, and practices (KAP) about the virus. In this sense, Health Education is an essential tool for dealing with the virus. The aim of health education is to educate individuals through educational, motivational, skill development, and awareness techniques, and an understanding of the main needs of KAP is essential for this. Many KAP studies were published during the COVID-19 pandemic, and the aim of the present study was to analyze these publications through a bibliometric study.

**Methods:**

A bibliometric analysis of the publications on KAP and COVID-19 was conducted in the Web of Science Core Collection database. The RStudio Bibliometrix and VOSviewer packages were utilized to analyze the scientific production, authors, citations, countries, publishers, journals, research areas, and keywords.

**Results:**

Of the 1,129 articles published, 777 were included in the study. The year with the most publications and citations was 2021. Three authors were underlined (all from Ethiopia), due to the number of articles published, the number of citations, and the collaboration networks established. As for the countries, most of the publications came from Saudi Arabia, while China obtained the most citations. PLOS One and Frontiers in Public Health published the most articles on the subject. The most frequent keywords were knowledge, attitudes, practices, and COVID-19. At the same time, others were identified based on the population group analyzed.

**Conclusion:**

This is the first bibliometric study on KAP and COVID-19. The significant number of publications identified on KAP and its relationship to the COVID-19 pandemic, in the span of only 3 years, indicates the increased interest in this area. The study provides relevant information to researchers who are approaching this subject for the first time. It is a useful tool that can stimulate new studies and collaborations between researchers from different countries, areas and approaches. At the methodological level, a step-by-step guide is provided for future authors who wish to perform a bibliometric analysis.

## 1. Introduction

COVID-19 is an infectious disease caused by the SARS-CoV-2 virus. The global pandemic it caused had not only health and social consequences, but also economic and environmental impacts (1, 2). For this, some studies (3) showed that the COVID-19 pandemic posed a global challenge for the achievement of the Sustainable Development Goals (SDG) with negative and positive correlations, and differences and inequalities among countries.

Considering the health aspects, and according to the World Health Organization (WHO) (4), the best type of prevention to stop its transmission was to be well informed about the disease and the propagation of the virus itself. Consequently, precise and up-to-date information interventions were indispensable for the population, to avoid disinformation and possible questionable practices (5). In this sense, within SDG 4, on Quality Education, we find Health Education (HE) (3), which is a fundamental tool for health literacy (6). HE helps individuals, professionals, organizations, and systems, to improve health through the empowerment of people in the making of informed decisions (7), and to facilitate changes in behavior (8). Also, it allows different manners of communication, and therefore, it is a versatile and adaptable method (9). Its aim is to educate people through educational, motivational, skill development, and awareness techniques (10). Thus, it is an essential element for facing and mitigating a worldwide pandemic such as the COVID-19 pandemic. However, the training must be massive and cover the entire social strata (11) in order to truly reduce health inequalities and improve the overall well-being of a community (12). Vamos and McDermott (7) detailed three important conditioning factors for the development of health literacy: (i) people must clearly know the reason for the program, (ii) they must have the resources and systems of support, and (iii) receive positive re-enforcement to maintain these actions.

Along this line, before performing an intervention or implementing a program, HE can include an evaluation of the knowledge, attitudes, and practice (KAP) of the target population (6). This will make it possible to implement effective interventions (13) adapted to a population or to new situations, such as the one created by COVID-19 (14). In this way, beneficial behaviors will be adopted in order to achieve a healthy way of life (15, 16). In this sense, and after more than 2 years of living with the pandemic, many studies (17–19) have focused on analyzing the KAP of the population to be able to design HE interventions. Given the above, the moment is ripe for analyzing, understanding, and observing the trend of the available scientific literature on the subject. Therefore, the following research question was posed: “What was the trend of scientific production on KAPs in the general population during the COVID-19 pandemic? In this regard, one of the best ways to do this is through the use of bibliometric analysis. Etymologically, the term “bibliometry” is composed of two words “biblio,” which means “book” in Greek, and “Metricus,” which refers to “measurement” in Greek (20). One of the pioneers of bibliometrics was Alan Pritchard in 1969, who used this term for the first time to refer to a new discipline that studies scientific production. Pritchard (21) defined bibliometrics as “the application of mathematics and statistical methods to books and other media of communication.” Even though, it was Garfield (22) who suggested that Science Citation Index (SCI) “would clearly be particularly useful in historical research, when one is trying to evaluate the significance of a particular work and its impact on the literature and thinking of the period” and who, a few years later, introduced the use of citation analysis and impact factor as tools for evaluating journals (23, 24).

According to a recent historical bibliometrics analysis (25), bibliometrics are becoming popular and increasing in medical research. Furthermore, in health research, bibliometrics are useful methods for analyzing the development of knowledge production (25). Thus, this is a type of scientometric study that utilizes mathematical and statistical data to map information, and can therefore be used to analyze all types of documents in order to understand publication trends and patterns (26). Its main objective is the quantitative analysis of a large number of articles and massive data, and can therefore have a great impact on research (27).

For all of these reasons, the present study is the first bibliometric study that is currently known that offers a descriptive and quantitative view of the articles published on KAPs of the general population during the COVID-19 pandemic. In this way, the study will allow other researchers to obtain a broad view on the publications on the subject, including the research trends and the most influential subjects, which could have repercussions in the future. Therefore, the objective of this bibliometric study was to quantitatively analyze scientific production on knowledge, attitudes, and practices of the general population during the COVID-19 pandemic.

## 2. Methods

Given that a guide that detailed the methodological steps for performing a bibliometric analysis was not found, the methods used in the present study were structured in the following manner: six steps were developed that were organized into three stages described by Fauzi (28): (1) data collection, (2) screening, and (3) analyzing the data. The literature research and identifying relevant studies steps were added to the first stage. In the second stage, two steps were taken, eligibility criteria and study selection and data collection. For the last stage, the grouping of the main analytical techniques described by Donthu et al. (27), performance analysis and science mapping, was utilized.

### 2.1. Stage 1: data collection

#### 2.1.1. Literature research

The search was performed in the Web of Science Core Collection (WoSCC) on August 8th, 2022. This is an international database that contains more than 61 million records from around the world (29), and it includes high-impact journals (30, 31), offers high quality articles, and allows their export for a bibliometric study (27, 32).

Given that the object of the research was to study the KAP during the COVID-19 pandemic, a search string was created which included the following keywords: “Knowledge,” “attitude,” “practise OR practice,” and “COVID-19 OR SARS coronavirus 2.” To focus the search, it was limited to “topic” instead of “all fields,” which means that the keywords were limited to those that appeared on the title, abstract, or keywords.

#### 2.1.2. Identifying relevant studies

To identify the relevant articles, and to include them in the results, inclusion criteria were defined centered on population, concept, and context (33) (Table 1).

**Table 1:** Inclusion criteria according to population, concept and context items.

Items	Description
Population	General population, without delimiting by sex, age, socioeconomic level, profession, cultural aspects, health situation, among others.
Concept	Knowledge, Attitudes and Practices (KAP)
Context	COVID-19 pandemic

The search strategy did not place limits on language or publication date. As for the type of publication, only journal publications and early access articles were included, while other formats (letters, meeting summaries, reviews, etc.) were excluded. Finally, articles that focused on specific population groups, for example, health professionals, were not excluded, as the objective of the present study was to discover the KAP in the general population.

### 2.2. Stage 2: screening

#### 2.2.1. Eligibility criteria

To delimit the quality of the articles, the “type of document” filter was utilized. Then, during the review according to title and abstract, the “type of document” was again utilized to meet the inclusion and exclusion criteria.

#### 2.2.2. Study selection and data collection

The selection of articles according to their abstract and titles was independently conducted by two of the researchers (LS and CC), and any discrepancy was solved by a third reviewer (TB).

The Preferred Reporting Items for Systematic Reviews and Meta-Analyses (PRISMA) methodology was followed for the article selection process (34).

### 2.3. Stage 3: analyzing the data

#### 2.3.1. Performance analysis

First, a descriptive bibliometric analysis was performed, also called performance analysis by Donthu et al. (27), starting with the Web of Science tool (WoS). The following information was extracted: (i) publication-related metrics: total publications (TP), number of contributing authors (NCA), number of active years of publication (NAY); (ii) citation-related metrics: local citations (LC) and global or total citations (GC or TC); and (iii) citation-and-publication-related metrics: H-Index. Also, additional information from the journals was utilized, such as: category, publisher, and impact factor (IF) according to the Journal Citation Report (JCR).

LC and TC were differentiated, the LC metric will sometimes indicate that an author or article from the collection is cited by another article in the same collection (35), and in contrast, the GC or TC metrics refer to the authors or articles most cited in WoS (36, 37).

In addition, the articles included were manually reviewed by two researchers (CC and LS) to extract the type of population studied, with the population classified as: health care workers (HCW)/healthcare providers, patients, students, and other population (all the articles that were not specifically directed to any of the first three groups were included in this last group).

#### 2.3.2. Science mapping

Lastly, and after the performance analysis and classification, the science mapping of the results was performed through the extraction of the information in two different formats: one for analysis with the R software (version 2022.07.0, RStudio Team, Boston, MA, United States) (38), and another for VOSviewer (version 1.6.17, Leiden University Center for Science and Technology Studies, Leiden, The Netherlands) (39). In Rstudio, the “Bibliometrix 4.0” package was exclusively utilized (35).

For these analyses, the following variables were utilized: citations, affiliations, countries, and keywords (considering the Keywords Plus and the author’s keywords together and separately). The Keywords Plus correspond to words identified by WoS in the titles of the articles (40), while the author’s keywords are defined by the authors of the publication (41).

Different analyses were conducted with respect to science mapping. First, a citation analysis was performed (relationships between publications and most influential publications) (27). In second place, a co-authorship analysis through Bibliometrix (40) was performed, which identified the social interaction between authors, and author affiliations (countries) (27). Then, a co-word analysis was performed (existing relationships between topics), as well as the analysis of co-occurrence of the keywords, through the use of the two software programs mentioned above: VOSviewer and Bibliometrix. In fourth place, different co-occurrence analyses were performed of the total keywords with VOSviewer, of the total database (at the general level and as a function of the publication date), and lastly, of the subgroups generated (HCW, patients, students and other population). In all cases, the value of the frequencies were adjusted and modified to obtain networks that were visually similar with respect to the number of words represented. And lastly, a Thematic Evolution Analysis was performed based on the connections between the author’s keywords (42).

The interaction analyses performed (authors, countries, and keywords) allowed us to evaluate their more frequent relationships (43), and provided us with a list of connections between them and their resulting network. The network of connections was represented through different-sized circles or nodes (for each author, country, or keyword), which represented their frequency, and lines of different thicknesses that connect the nodes, representing the intensity of occurrence (44, 45). The greater the frequency, the greater the correlation, and an increased probability of belonging to the same cluster (46).

## 3. Results

**Figure 1:**
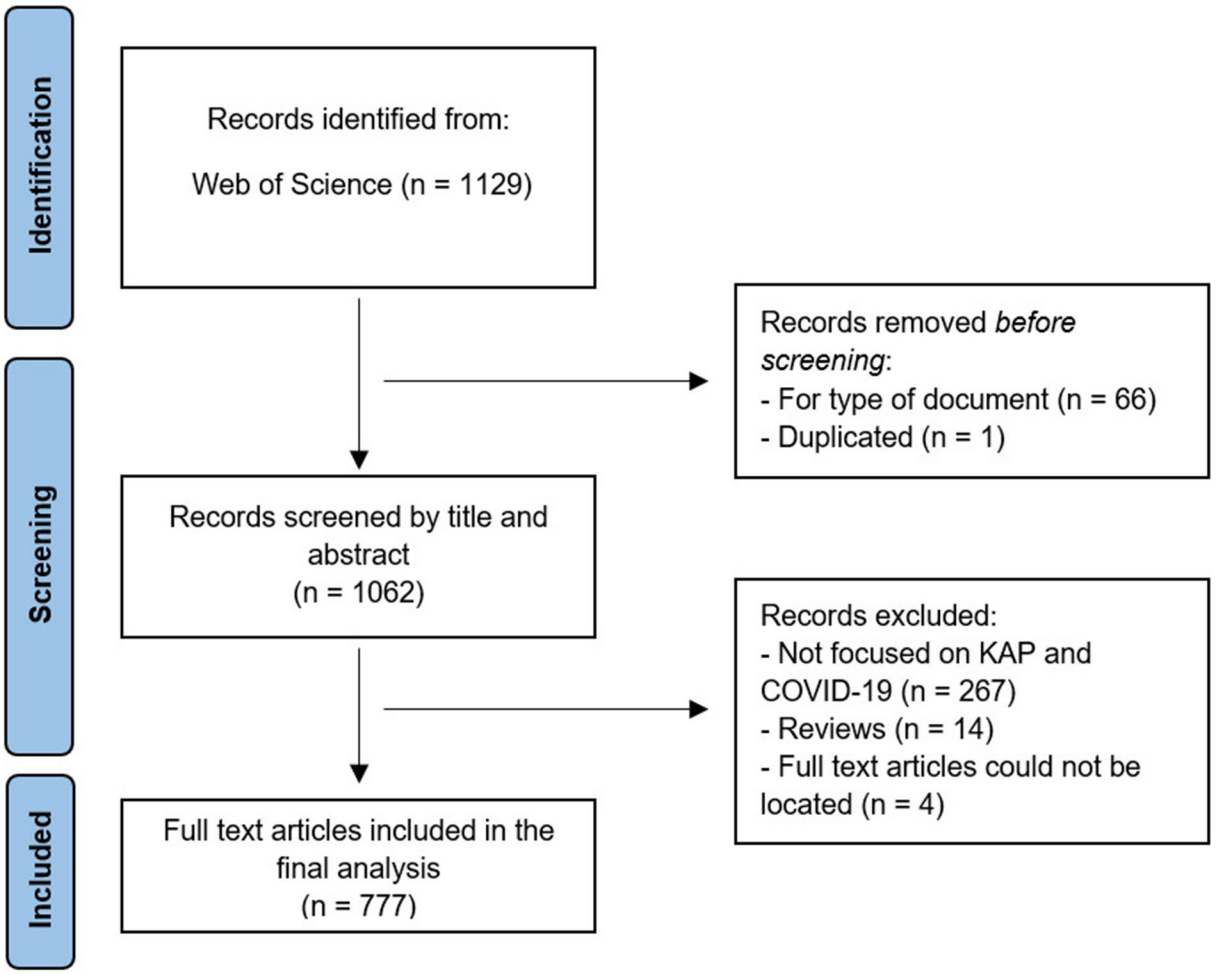
Flowchart of the results of the search according to the PRISMA standard (adapted version).

In total, 1,129 articles were identified, and after the elimination of duplicates and the application of the filters according to the type of document, 1,062 articles were analyzed with respect to the title and abstract, considering the inclusion and exclusion criteria. Finally, 777 articles were included (Figure 1).

### 3.1. General characteristics of the bibliometric analysis

**Figure 2:**
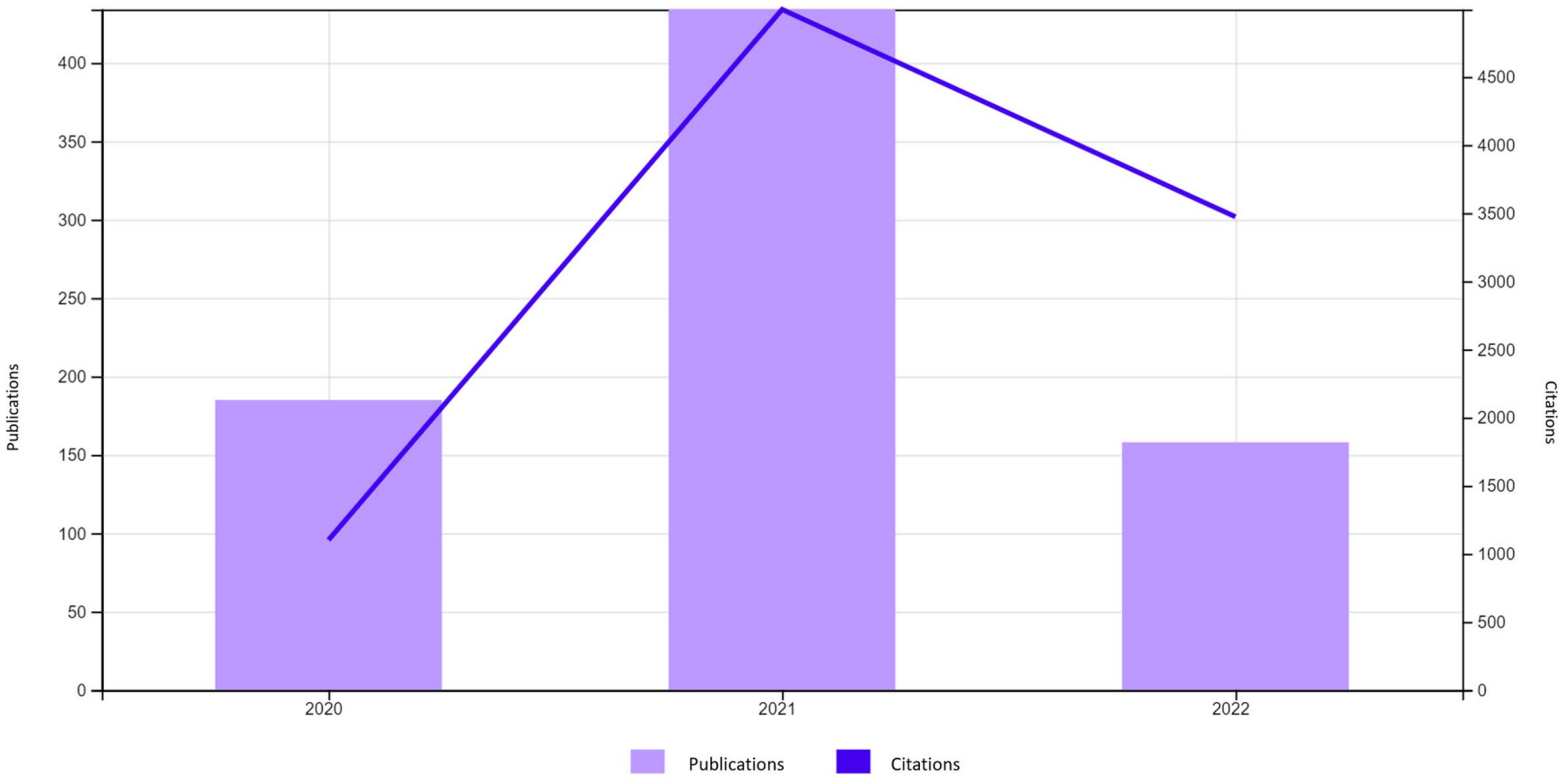
Number of publications and citations per year. Source: Web of Science.

With respect to the NAY, all the articles were published between the years 2020 and 2022 (Figure 2), with the largest number of publications (~56%) and citations found in 2021.

Through the citation report (“Analyze results”), obtained directly from WoSCC, and a manual review of the publications to identify the population studied, we identified the general characteristics of the articles included in the bibliometric analysis (Table 2). The results on language used indicated that most of the publications were written in English, and as for the population studied, slightly more than half of the publications were on specific population groups.

**Table 2:** Summary of key characteristics of included articles.

Classification	Articles (*N* = 777) No. (%)
**Language**	
English	769 (98.97)
Spanish	6 (0.77)
French	1 (0.13)
German	1 (0.13)
**Population**	
Health care workers (HCW)/healthcare providers*	253 (32.56)
Students**	121 (15.57)
Patientsˆ	73 (9.39)
Other populationˠ	330 (42.47)

* Health care workers (HCW)/healthcare providers included: physicians, nurses and midwives, nursing assistants, dentists and dental assistants, pharmacists, physiotherapists, psychologists, ophthalmologists and medical laboratory professionals.

** Students included: high school students/middle school students, university undergraduates/university students/college students.

ˆPatients included: people living with HIV/AIDS, people with rheumatoid arthritis, cancer, gynecological oncology, COVID-19, hypertension, heart disease, chronic disease, dermatological disease, psychiatric disease or spinal cord injury; people admitted to hospitals, psychiatric hospitals, or diagnosed with a previous myocardial infarction; and people visiting dental institutions, attending a family medicine clinic, outpatient screening visits for COVID-19, visiting the outpatient service or visiting eye hospitals, pregnant women and women seeking fertility treatment. ˠIn the Other population group, all the articles that were not directed specifically to the three previous groups were placed here (i.e., adult population, older adult people, rural population, urban residents, restaurant customers, religious, travelers, etc.).

### 3.2. Authors

From the 777 articles included, a total of 4,728 authors were identified. The authors with the highest number of published documents were: M. Adane (*n* = 7), G. Berihun (*n* = 6) and M. Baig, D. Teshome, and Z. Walle (all of them with 5 articles). Nevertheless, when considering another measurement, the five authors with the highest impact (all of them with an H-Index of 4) were: Teshome, H. Sacre, A. Khaled, Y. Zhou and M.M. Rahman (with 5, 4, 3, 3, and 2 articles, respectively).

In addition, the results indicated that 27.93% of the authors had written a publication with other international authors (international co-authorships). Of these, G. Berihun established the most collaboration networks, followed by L. Berhanu, D. Teshome, M. Adane, Z. Walle and M. Abebe.

### 3.3. Most cited documents

Table 3 summarizes the characteristics of the ten most-cited articles. Zhong et al. (52), with the article “Knowledge, attitudes, and practices towards COVID-19 among Chinese residents during the rapid rise period of the COVID-19 outbreak: a quick online cross-sectional survey,” sets itself apart from the rest with respect to the number of citations, as it accumulated 1,090 TC. This article was published in the international journal of Biological Science. With respect to the population studied in the articles, half of them were centered on the “other population” group (47, 49–52), four towards health professionals (48, 53–55), and one on patients (individuals with chronic diseases) (56).

**Table 3:** Top 10 cited documents.

N°	Authors (publication year) (reference)	Title	Population group classification (population of the study, n)	Citations (Global citations)	Journal
1	Zhong et al. (2020) (43)	Knowledge, attitudes, and practices towards COVID-19 among Chinese residents during the rapid rise period of the COVID-19 outbreak: a quick online cross-sectional survey	Residents (Chinese residents, *n* = 6.910)	1,090	International Journal of Biological Science
2	Azlan et al. (2020) (44)	Public knowledge, attitudes and practices towards COVID-19: a cross-sectional study in Malaysia	Residents (Malaysian residents, *n* = 4.850)	375	PLoS One
3	Zhang et al. (2020) (47)	Knowledge, attitude, and practice regarding COVID-19 among healthcare workers in Henan, China	Health care workers (doctors, nurses, and paramedics, *n* = 1.357)	304	Journal of Hospital Infection
4	Al-Hanawi et al. (2020) (45)	Knowledge, attitude and practice toward COVID-19 among the public in the Kingdom of Saudi Arabia: a cross-sectional study	Residents (general population of Saudi Arabian, *n* = 3.388)	299	Frontiers in Public Health
5	Wolf et al. (2020) (48)	Awareness, attitudes, and actions related to COVID-19 among adults with chronic conditions at the onset of the United States outbreak a cross-sectional survey	Patients (United States adults aged 23 to 88 years living with 1 or more chronic conditions, *n* = 630)	287	Annals of Internal Medicine
6	Saqlain et al. (2020) (49)	Knowledge, attitude, practice and perceived barriers among healthcare workers regarding COVID-19: a cross-sectional survey from Pakistan	Health care workers (doctors, pharmacists and nurses, *n* = 414)	225	Journal of Hospital Infection
7	Olum et al. (2020) (50)	Coronavirus disease-2019: knowledge, attitude, and practices of health care workers at Makerere University Teaching Hospitals, Uganda	Health care workers (nurses, midwives, internship doctors, medical officers, senior house officers, and specialists, *n* = 581)	213	Frontiers in Public Health
8	Khader et al. (2020) (51)	Dentists’ awareness, perception, and attitude regarding COVID-19 and infection control: cross-sectional study among Jordanian dentists	Health care workers (dentists, *n* = 700)	179	JMIR Public Health and Surveillance
9	Ferdous et al. (2020) (46)	Knowledge, attitude, and practice regarding COVID-19 outbreak in Bangladesh: an online-based cross-sectional study	Residents (Bangladeshi residents aged 12–64 years, *n* = 2.017)	166	PLoS One
10	Reuben et al. (2021) (52)	Knowledge, Attitudes and Practices Towards COVID-19: An Epidemiological Survey in North-Central Nigeria	Residents (residents of north-central Nigeria, *n* = 589)	153	Journal of Community Health

### 3.4. Countries

The results on the origin of the articles showed that the country with the most citations was China, followed by Saudi Arabia, United States, Pakistan, and Ethiopia. Nevertheless, according to the number of documents, Saudi Arabia and India had the most publications, followed by Ethiopia and the United States (Table 4).

**Table 4:** Country ranking according to number of citations and number of articles published.

Ranking position by number of citations	Country	No. of citations	Ranking position by number of papers	No. of papers
1	Peoples R China	2019	5	65
2	Saudi Arabia	875	1	113
3	USA	803	4	69
4	Pakistan	561	6	63
5	Ethiopia	536	3	69
6	India	531	2	110
7	Malaysia	468	7	39
8	Jordan	462	10	33
9	Nigeria	295	13	26
10	Bangladesh	282	11	28

Considering the institutions with the most influence (those with more than 20 articles published in the three-year period analyzed, 2020–2022), these were: University of Gondar (*n* = 39) located in Ethiopia, King Saud University (*n* = 28), from Saudi Arabia, and the Egyptian Knowledge Bank (EKB) (*n* = 23) located in Egypt.

**Figure 3:**
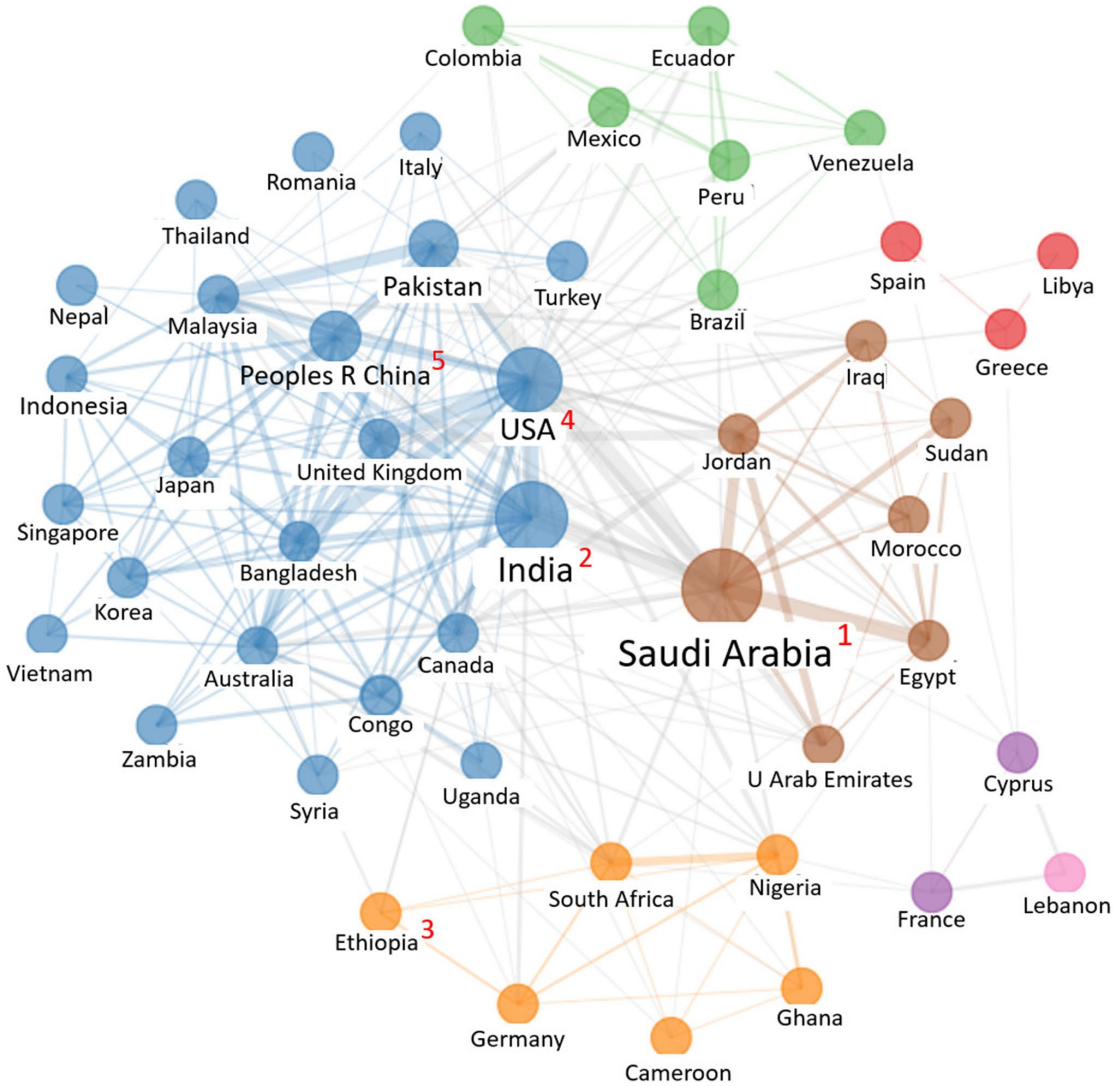
Network visualization map of co-authorship country and the top five countries with the highest number of publications (this is indicated as superscript numbers in red, from 1 to 5).

The existing collaboration between countries was also analyzed. Figure 3 shows that the countries with the most collaborations, resulting in the highest number of contact networks, were Saudi Arabia, India, the United States, People’s Republic of China, and Pakistan. More specifically, it was observed that the collaboration networks could be broken down into seven clusters: (i) blue, composed of 23 countries, three of which were in the top 5 in number of publications (India, the United States, and People’s Republic of China); (ii) brown: composed of 7 countries, among which we find Saudi Arabia, the country with the highest number of publications; (iii) orange: composed of 6 countries, one of which is Ethiopia (third-highest country in number of publications); (iv) green: composed of 6 countries; (v) purple: composed of France and Cyprus; and (vi) pink, with a single country, Lebanon.

### 3.5. Publishers, journals and research areas

The publishers with the highest number of publications were the Multidisciplinary Digital Publishing Institute (MDPI) (*n* = 83, 10.68%), Springer Nature (*n* = 71, 9.14%), Dove Medical Press Ltd., (*n* = 63, 8.11%), Public Library of Science (*n* = 61, 7.85%) and Frontiers Media S.A. (*n* = 60, 7.72%), with the rest of the publishers having less than 50 publications each.

**Figure 4:**
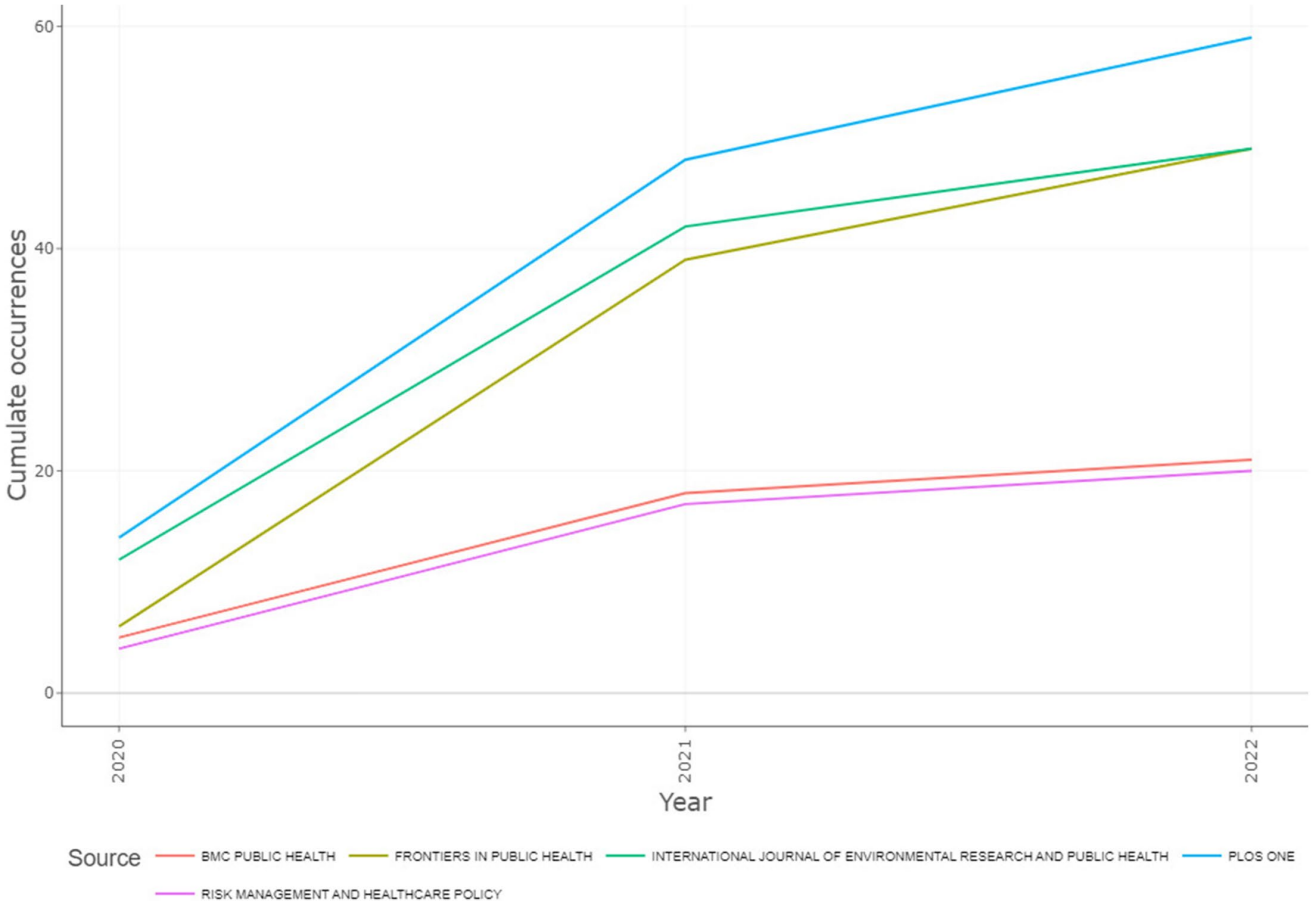
Source Growth by cumulate occurrences.

The five journals with the highest number of publications were PLOS ONE (59, 7.59%), Frontiers in Public Health (49, 6.30%), International Journal of Environmental Research and Public Health (49, 6.30%), BMC Public Health (21, 2.70%), and Risk Management and Healthcare Policy (20, 2.57%). The journal with the greatest growth since 2020, with respect to the total number of publications was Frontiers in Public Health (Figure 4).

As for the journals with the highest impact, these were: PLOS ONE (786 LC), Lancet (576 LC), International Journal of Environmental Research and Public Health (508 LC), Frontiers in Public Health (447 LC), and The New England Journal of Medicine (383 LC). The journals that were in the top 5 in the number of publications, such as BMC Public Health and Risk Management and Healthcare Policy, were found in the seventh (356 LC), and fortieth positions (67 LC), respectively. However, when considering other measurements of impact, such as TC and H-Index, the results were different. The top five sources with the highest TC were: PLOS ONE (1,205 TC), International Journal of Biological Science (1,090 TC), Frontiers in Public Health (795 TC), International Journal of Environmental Research and Public Health (628 TC), and Journal of Hospital Infection (529 TC). Two of the journals that were found in the top 5 in the number of publications, occupied lower positions with respect to the number of TC: BMC Public Health was found in the seventh position, with 410 TC, and Risk Management and Healthcare Policy was in the eleventh position, with 132 TC. As for the H-Index, the order of the journals with the most impact were: PLOS ONE (16 H-Index), International Journal of Environmental Research and Public Health (11), BMC Public Health (10), Journal of Community Health (10), and Frontiers in Public Health (9), with the Risk Management and Healthcare Policy journal found in the eight position, with an H-Index of 7.

If we analyze the impact of the research area or category, as a function of the number of publications, it was observed that the Public, Environmental & Occupational Health category occupied the first position (*n* = 264, 33.98%), followed by Medicine, General and Internal (*n* = 117, 15.06%), and Health Care Sciences and Services (*n* = 84, 10.81%). According to the Journal Citation Reports, each of these categories was comprised by 160, 330 and 160 journals, respectively. As for the WoS index, 474 (61.00%) were found in the Science Citation Index Expanded (SCI-Expanded or SCIE), 287 (36.94%) in the Social Science Citation Index, and 265 (34.11%) in the Emerging Sources Citation Index (ESCI).

Lastly, as a summary, the journals that were found in the top 5 positions of any of the four impact indicators utilized, were analyzed (record count, no. of LC, TC and H-Index) (Table 5). A total of ten journals were identified. It should be highlighted that they originated from different publishers, as most of them belonged to a different publisher (except two of them, which belonged to Elsevier). With respect to category, half of the journals were classified in the Public, Environmental and Occupational Health category, and the SCIE publication.

**Table 5:** Characteristics of the journals with the most relevance in different classifications (by record count, no. of local citations, total citations and H-Index).

Publishers	Journal	TOP 5				JIF (2021)	JIF without self-citations (2021)	Edition	Research area or category	JIF rank and quartile (2021)
		Record count	No. of local citations	Total citations	H-Index					
Public Library of Science (PLoS)	PLoS One	x	x	x	x	3.752	3.608	SCIE	Multidisciplinary Science	29/73 Q2
								SCIE	Biology	NA
Frontiers Media S.A.	Frontiers in Public Health	x	x	x	x	6.461	6.122	SSCI	Public, Environmental and Occupational Health	18/182 Q1
								SCIE	Public, Environmental and Occupational Health	37/210 Q1
MDPI	International Journal of Environmental Research and Public Health	x	x	x	x	4.614	3.994	SCIE	Environmental Science	100/279 Q2
								SCIE	Public, Environmental and Occupational Health	71/210 Q2
								SSCI	Public, Environmental and Occupational Health	45/182 Q1
BioMed Central Ltd.	BMC Public Health	x			x	4.135	3.944	SCIE	Public, Environmental and Occupational Health	83/210 Q2
Dove Medical Press Ltd.	Risk Management and Healthcare Policy	x				2.853	2.688	SCIE	Health Care Sciences and Services	64/109 Q3
								SSCI	Health Policy and Services	43/88 Q2
Elsevier	Lancet		x			202.731	201.484	SCIE	Medicine, General and Internal	1/172 Q1
Massachusetts Medical Society	New England Journal of Medicine		x			176.079	175.310	SCIE	Medicine, General and Internal	2/172 Q1
Ivyspring International Publisher	International Journal of Biological Science			x		10.750	10.626	SCIE	Biochemistry and Molecular Biology	28/296 Q1
Elsevier	Journal of Hospital Infection			x		8.944	8.579	SCIE	Infectious Diseases	18/94 Q1
								SCIE	Public, Environmental and Occupational Health	18/210 Q1
Springer Science+Business Media	Journal of Community Health				x	4.371	4.325	SSCI	Health Policy and Services	15/88 Q1
								SSCI	Public, Environmental and Occupational Health	51/182 Q2

JIF, journal impact factor; SCIE, science citation index expanded; SSCI, social sciences citation index; NA, not available.

### 3.6. Keyword co-occurrence analysis

In general, 443 keyword Plus and 1,117 author’s keywords were found in the 777 publications analyzed in the present review.

**Figure 5:**
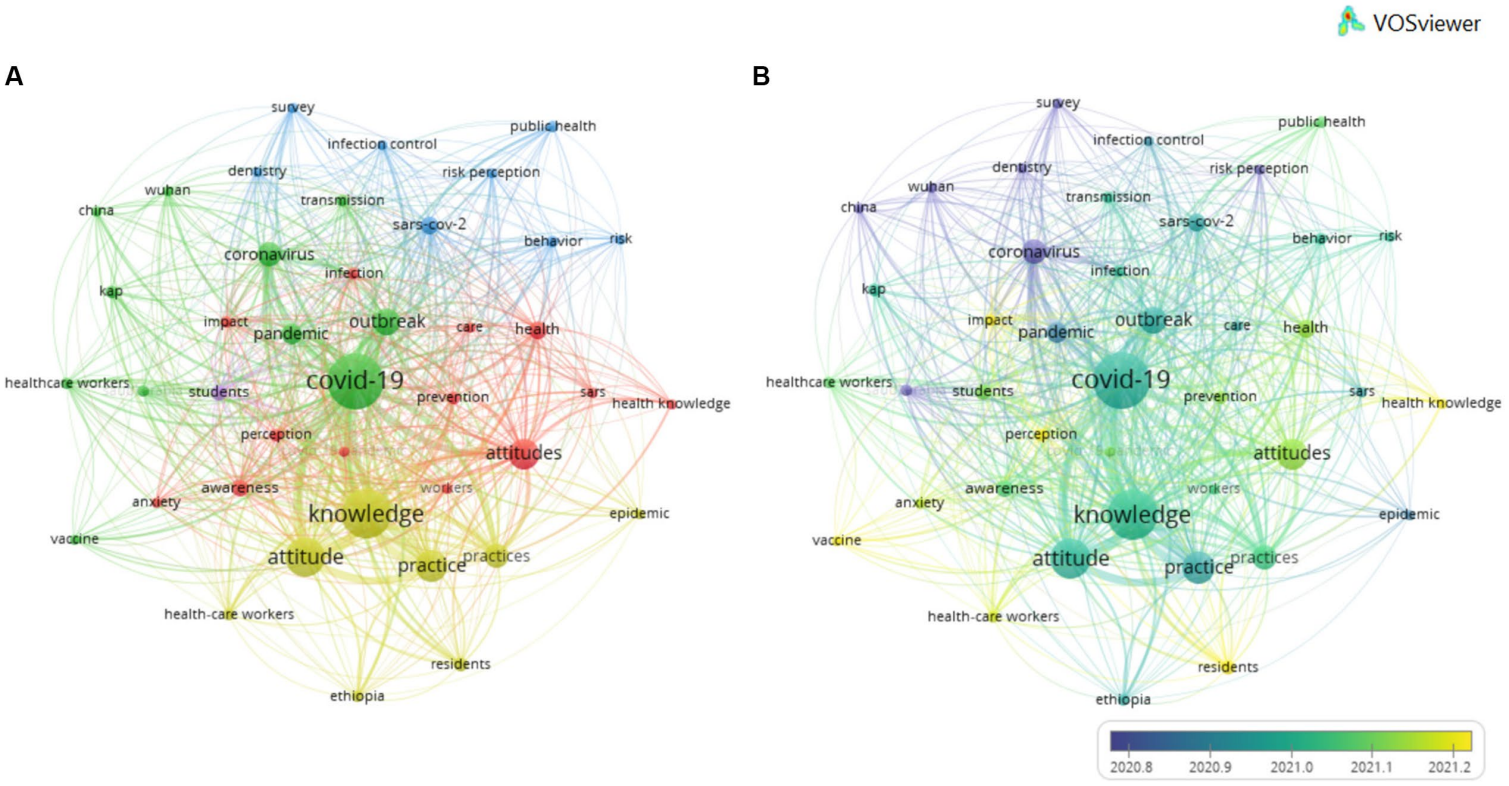
Network map of 15,151 keywords with frequency more than 17. The network on the left **(A)** shows the clusters created, and that on the right **(B)** shows the same results as a function of the publication date.

When considering the total number of keywords (including the Keywords Plus and the Author’s keywords) of the publications, and when they were examined through co-occurrence analysis (Figure 5A), it was observed that the most frequent were: COVID-19 (526 occurrences), knowledge (416), attitude (260), practice (175) and attitudes (161). Also, a total of 5 clusters were found. The cluster with the most items (#1) contained: anxiety, attitudes, awareness, care, COVID-19 pandemic, health, health knowledge, impact infection, perception, prevention, Sars and workers. The second cluster (#2) was composed by: China, coronavirus, COVID-19, healthcare workers, KAP, outbreak, pandemic, Saudi Arabia, transmission, vaccine and Wuhan. The third cluster (#3) contained: behavior, dentistry, infection control, public health, risk, risk perception, SARS-COV-2 and survey. The fourth cluster (#4): attitude, epidemic, Ethiopia, health-care workers, knowledge, practice, practices and residents, and lastly, the fifth cluster (#5) was composed by a single word, students.

If these networks were analyzed with respect to the year of publication, it was observed that the most frequent keywords at the start of the pandemic (in 2020) were: coronavirus, China, Wuhan, dentistry and survey. The trend towards 2021 showed keywords such as: pandemic, practice, covid-10, knowledge, attitude, and outbreak. Lastly, after 2021, the keywords were: health-care workers, anxiety, impact, health knowledge, vaccine, perceptions and residents (Figure 5B).

**Figure 6:**
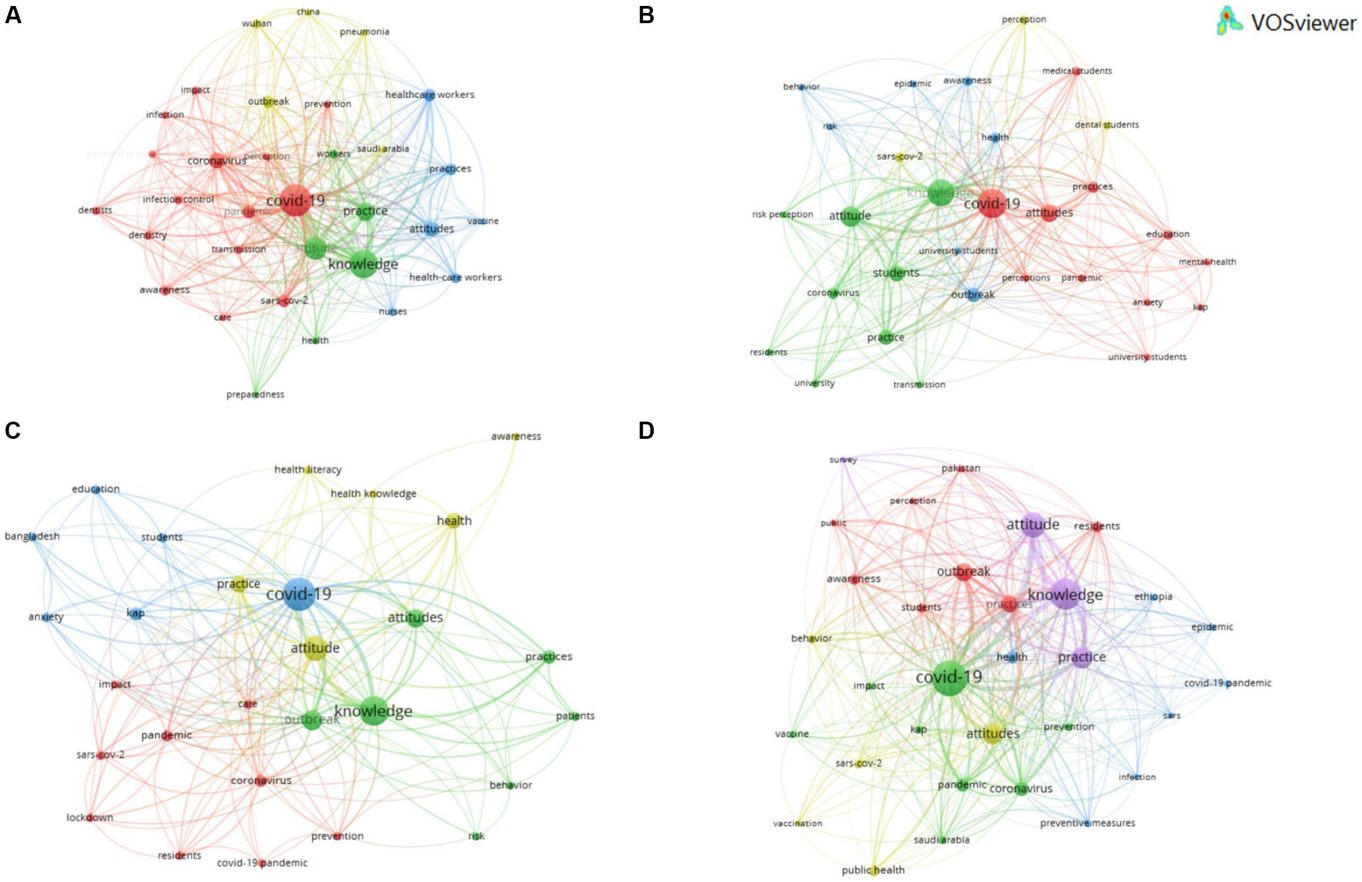
Network map as a function of the grouping of the articles according to type of population: **(A)** Health care workers (619 keywords with a frequency of more than 8); **(B)** students (368 keywords with a frequency of more than 5); **(C)** patients (276 keywords with a frequency of more than 3); and **(D)** other population (792 keywords with a frequency of more than 8)/.

When the same analysis was conducted, but this time with each of the population groups generated (HCW, patients, students, and other population), it was observed that the most frequent keywords in all of them were: COVID-19, knowledge and attitude (Figure 6).

The first group shown corresponded to the HCW, and four defined clusters were observed (Figure 6A). Some keywords from the clusters stood out: (#1) dentistry, dentists, personal protective equipment and transmission; and also (#3) nurses and vaccine. The group corresponding to the students was differentiated between four clusters (Figure 6B), highlighting: (#1) anxiety, medical students, mental-health, university students; (#2) university; and (#4) dental students. From the analysis of the patient group (Figure 6C), the following were observed: (#1) anxiety and care; and (#2) health literacy. With respect to the last group analyzed, “other population,” five clusters were generated (Figure 6D) with words such as: (#1) residents and students; (#2) vaccine; (#3) Ethiopia and preventive measures; (#4) behavior, public health and vaccination; and (5#) attitude, knowledge, practice and survey.

**Figure 7:**
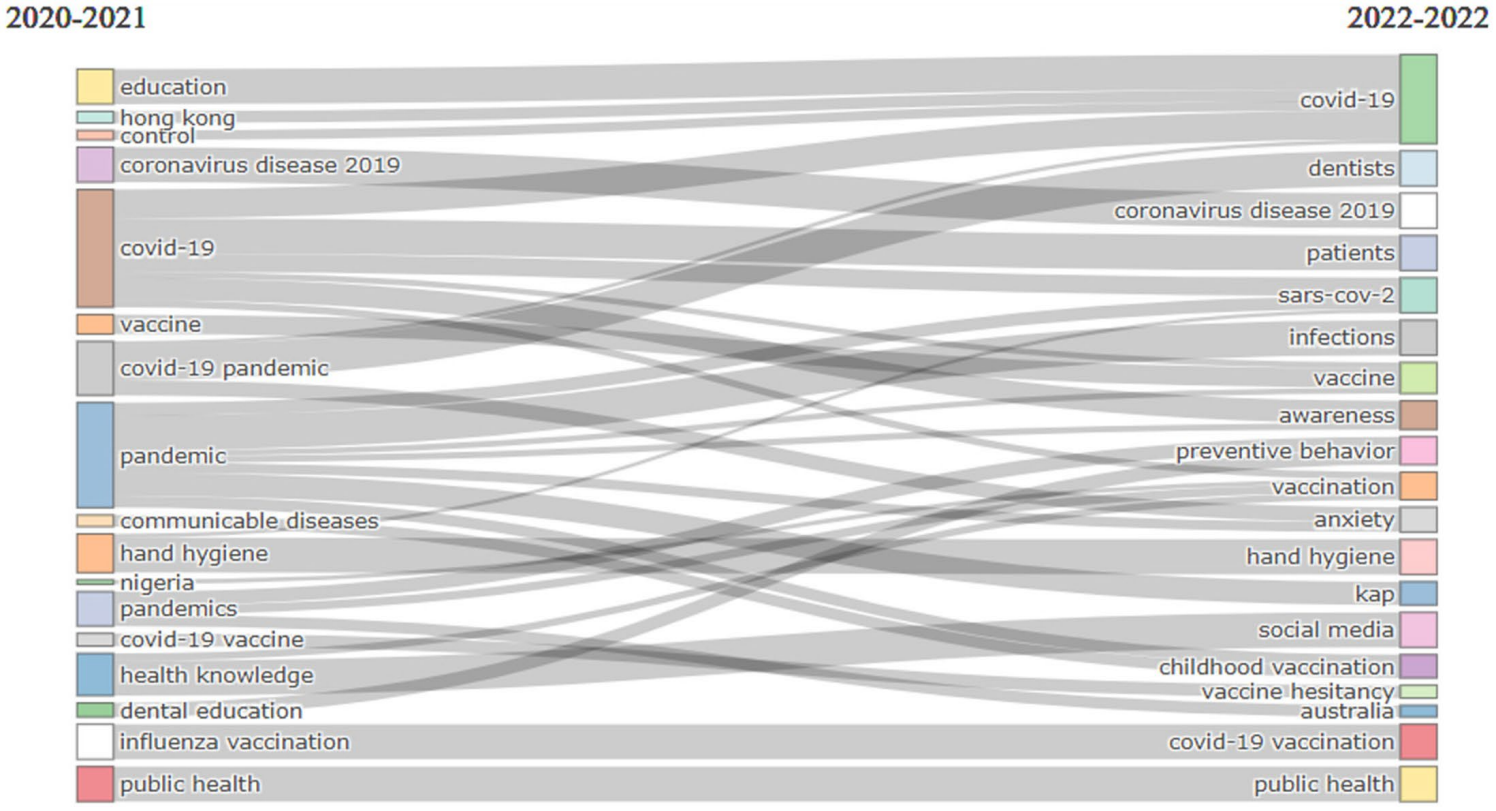
Author’s keywords thematic evolution between 2020 and 2022.

Lastly, the author’s keywords were analyzed independently throughout the 3 years studied (2020–2022). With respect to the trends, the only word that was common throughout the years as a trending topic was “face mask.” Words such as “COVID-19,” “knowledge” and “attitude” were the most frequent (515, 354 and 224 respectively) in these 3 years (specifically in 2021). However, if the changes in the theme of the author’s keywords throughout these 3 years (with a cutoff year of 2021) (Figure 7) are shown, it was observed that 2020 contained general words such as COVID-19 (coronavirus disease 2019, COVID-19 pandemic, pandemics and pandemic), and 2022 contained more specific author’s keywords, such as preventive behavior, anxiety and hand hygiene.

## 4. Discussion

As far as we know, this is the first bibliometric article that analyzes the scientific production on KAP and COVID-19. Although other bibliometric analyses exist, on COVID-19 in general (32, 57, 58), or more specific ones such as vaccination (59), other personal protection equipment (37), e-learning (24) or on the integration of digital technologies and public health to combat COVID-19 (60), none included a specific bibliometric analysis on KAP and COVID-19. Therefore, the present bibliometry has contributed towards the understanding of the trends and patterns of the publications on the subject, through a descriptive analysis of the most-cited articles, and the countries with the most citations and articles, and the journals in which they were published. On the other hand, it has provided information on the co-authorships and information on the most prolific authors, and it also shows the results of an analysis of co-occurrence of the keywords utilized. Also, given that a guide was not available which described the methodological steps taken, a detailed proposal was described through the addition of structures from other authors (27, 28). Therefore, this study can be utilized by other researchers who want to perform a bibliometric analysis.

### 4.1. General characteristics of the bibliometric analysis

Given that COVID-19 emerged in December, 2019, publications were only found starting in the year 2020. In this short period of 3 years (2020–2022), 777 original articles were published on KAP and COVID-19. The highest number of publications and citations appeared in 2021, about a year after the start of the worldwide pandemic caused by COVID-19. The same trend was observed with respect to scientific production in other bibliometric studies (58, 59). However, some did not specify the years (28, 37, 61), or they studied the coronavirus respiratory syndrome (SARS-CoV) and COVID-19, for which scientific articles were found as far back as 2003 (57).

When analyzing the target audience of the articles, after which they were distributed into different groups, it was observed that more than half of the publications were directed to specific groups. This could be because health professionals, students (especially at the university level and in the area of health), and patients (for example, with chronic pathologies or currently under treatment), were the most affected by the pandemic (18, 62, 63). In fact, four out of the ten most-cited articles were directed towards health professionals (48, 53–55). However, older individuals and children, who are also very vulnerable groups that suffered from the impact of the pandemic, were not identified in the articles studied.

### 4.2. Authors and most cited documents

The most prolific authors were M. Adane and G. Berigun. This result coincided with the finding that these two authors had the most collaborations, and shared five of their publications, all of which were centered on Ethiopia (64–68). As for their affiliation, both were part of the Department of Environmental Health (Wollo University, Dessie, Ethiopia).

In fact, the most prolific authors did not coincide with those who had the highest impact, except for D. Teshome (also from Wollo University, Dessie, Ethiopia), who had a high H-index, and who took third place in scientific production with five publications. Also, this author shared four of the articles with M. Adane or G. Berihun (66–69). The co-authorships between D. Teshome, M. Adane and G. Berihun, allowed them to be defined as the three authors with the most collaboration networks.

In spite of this, the most-cited article came from Zhong et al. (52), published only 3 months (March, 2020), after the worldwide emergency caused by COVID-19 (December, 2019). This article was written by seven authors, and none of them were found among the most-cited or with the highest impact according to their H-Index. In reality, they only published this article among the 777 included in the present bibliometric study.

As for the number of citations of the articles, the highest number of citations came from the nine articles published in 2020, with this publishing speed allowing them to be cited in the following 2 years by articles that studied the same subject.

### 4.3. Countries

As for the countries with the most publications, Saudi Arabia and India had the most publications and collaborations. However, China had the highest number of citations, with a great advantage over the second-highest country, Saudi Arabia, although it was also found in fifth place with respect to the number of publications. This is because the first COVID-19 cases appeared in the Chinese city of Wuhan, and coinciding with Giannos et al. (70), another reason could be that China was the first country to take measures based on evidence to reduce the impact of COVID-19.

Although the authors with the most collaborations and publications or a high H-Index value were from Ethiopia, this country did not stand out with respect to cooperation with other countries, and was found in third place in the number of publications and in fifth place in the number of citations.

With respect to the collaboration networks between countries, some trends were observed. A greater collaboration was established between Latin American countries (Colombia, Ecuador, Mexico, Venezuela, Peru, and Brazil), Arabic countries (Saudi Arabia, United Arab Emirates, Egypt, Morocco, Sudan, Jordan, and Iraq), and between African countries (Ethiopia, South Africa, Nigeria, Ghana, and Cameroon). In contrast, no clusters were identified between EU countries; only small collaborations were found between Spain and Greece, and Romania and Italy. This indicates that there was little collaboration between the EU countries for the development of studies and publications about KAP and COVID-19.

### 4.4. Journals and research areas

To describe the quality of the journals, the five journals with the greatest impact as a function of four different quality indicators were analyzed, and only three of them, PLOS One, Frontiers in Public Health and International Journal of Environmental Research and Public Health, were found in the top five of the four indicators. These three journals were indexed in the SCIE publication, within which we found 61% of the articles included in the bibliometric study, in the Q1 and Q2 quartiles.

### 4.5. Keyword co-occurrence analysis

The bibliometric analysis of the different keywords revealed the existence of a great diversity of terms with a high co-occurrence, thus showing the heterogeneity of the concepts related with KAP and COVID-19. Among the words found, we also identified concepts related with populations that were more vulnerable to COVID-19, such as health professionals and students. In spite of this, it was surprising to find that we did not detect words such as patient, older adult, or pregnant women, with these populations also vulnerable to the disease (71–74). It is possible that on many publications, the pathology or condition (e.g., pregnancy, chronic disease, etc.) was indicated instead of the population group, resulting in their unintended concealment. In the present bibliometric study, we opted to go further and analyze the keywords according to the four population groups (i.e., healthcare workers, patients, students, and other population), with a different spectrum of keywords observed for each of them. Thus, in the group of articles that dealt with subjects related to health workers, terms such as nurses, dentists, personal protective equipment, vaccine or transmission were identified; these concepts are mostly related with virus transmission and collectives (nurses and dentists) with a greater exposure to the disease (75–77). As for the group of patients, the more common concepts were anxiety, care and health literacy. These concepts are closely related with the greater uneasiness experienced by this collective, due to the large lack of knowledge on this aspect, indicating the great need for health literacy. As for the group of students, the main keywords were related with concepts such as university, medical students and dentistry students. Also, two keywords were identified with emotional aspects such as anxiety and mental health, thus characterizing the association of this group with COVID-19 and the KAP, as also mentioned by other authors (78, 79). Lastly, for the other populations group, the more frequent terms were attitude, knowledge, practice and survey in a single cluster. This corroborates what was observed in the articles found associated to this group: all of them addressed KAP through surveys. Aside from these keywords, other high-frequency ones were found, such as: behavior, public health, vaccine or preventive measures, terms that addressed more heterogeneous concepts included in this disparate sector of the population.

Lastly, as for the evolution of the keywords in the short period of time analyzed, changes were observed in the usage trends of specific terms as the pandemic progressed. Thus, if at its start the terms were mostly associated with themes related to its origin (i.e., China and Wuhan) and infection control, towards the end of the pandemic, more publications were found about vaccines, anxiety, impact, or health knowledge. Other keywords that changed throughout the pandemic were related with prevention (i.e., face mask, hand hygiene, vaccine or vaccination) (80, 81), or with relating to others and obtaining information online (i.e., social media) (82). All of them are an indication of key aspects that were dealt with in a manner that was more or less specific, and which were maintained throughout the publications that dealt with the COVID-19 pandemic.

### 4.6. Limitations

As for the limitations of the present study, the first would be that the articles were obtained from a single database, WoSCC. However, this database is considered as the most important for bibliometric analyses (83). In second place, there was an inherent bias in the citations variable, as these vary every day, and also, it was expected that the older articles would have more citations (84). In third place, the keywords were not standardized before the co-occurrence analysis. This resulted in the appearance of some nodes that meant the same thing, for example, “attitude” and “attitudes,” so that the node was not larger with more co-occurrences. And lastly, as other authors suggested in their bibliometric study (57), experts were not utilized to analyze the evolution of the keywords and their repercussion in the different areas of research, and multidisciplinary experts from other affiliations were not contacted for providing strategic proposals for future studies.

## 5. Conclusion

This is the first bibliometric study that provides a detailed analysis of the scientific production on knowledge, attitudes and practices of the general population during the COVID-19 pandemic. The significant number of publications identified on KAP and its relationship to the COVID-19 pandemic, in the span of only 3 years, provides evidence of the increased interest in this area.

The information on the publications provided in the present article not only tracks the shift on the state of the subject, but also provides bibliographic information that is relevant to future studies. At the same time, the co-occurrence and subject evolution analyses contribute towards the identification of a conceptual structure, the thematic evolution of the research studies, and provides a prediction of the trends on which future studies should be conducted. The detailed analysis of the main keywords used by the authors, as well as the trends in their use, showed that the approach to KAPs was different according to the different population groups identified in the study. Therefore, the results provide an indication on the population groups in which greater research was conducted on the KAP during the COVID-19 pandemic, and the nature of the most relevant themes for each of these groups. In addition, the results provide relevant information to the researchers who approached this subject for the first time. Therefore, the information provided in this study is a useful tool that can stimulate new studies and collaborations between researchers from different countries, areas and approaches. Lastly, at the methodological level, it offers a step-by-step guide for future authors who want to perform a bibliometric analysis.

## Author contributions

LS-P and CCamí: conception, design and collection data. CCamí, JR, and TB: conceptualization. LS-P: methodology, software and data analysis. LS-P, CCamp, and TB: screening literature. LS-P, CCamí, and AE: writing-original draft preparation. TB and JR: writing-review and editing. All authors contributed to the article and approved the submitted version.

## Funding

This study was funded by the project IlerCOVID, which was supported by the Agency for Management of University and Research Grants of the Department of Research and Universities of Generalitat de Catalunya (file number 2020 PANDE 00124).

## Conflict of interest

The authors declare that the research was conducted in the absence of any commercial or financial relationships that could be construed as a potential conflict of interest.

## Publisher’s note

All claims expressed in this article are solely those of the authors and do not necessarily represent those of their affiliated organizations, or those of the publisher, the editors and the reviewers. Any product that may be evaluated in this article, or claim that may be made by its manufacturer, is not guaranteed or endorsed by the publisher.
